# Microbial eukaryotic diversity and distribution in a river plume and cyclonic eddy-influenced ecosystem in the South China Sea

**DOI:** 10.1002/mbo3.282

**Published:** 2015-08-12

**Authors:** Wenxue Wu, Lei Wang, Yu Liao, Bangqin Huang

**Affiliations:** 1 Key Laboratory of Coastal and Wetland Ecosystems, Xiamen UniversityXiamen, China; 2 State Key Laboratory of Marine Environmental Science, Xiamen UniversityXiamen, China; 3 Institute of Oceanography, National Taiwan UniversityTaipei, Taiwan

**Keywords:** 18S rDNA, mesoscale process, microbial eukaryote, South China Sea

## Abstract

To evaluate microbial eukaryotic diversity and distribution in mesoscale processes, we investigated 18S rDNA diversity in a river plume and cyclonic eddy-influenced ecosystem in the southwestern South China Sea (SCS). Restriction fragment length polymorphism analysis was carried out using multiple primer sets. Relative to a wide range of previous similar studies, we observed a significantly higher proportion of sequences of pigmented taxa. Among the photosynthetic groups, Haptophyta accounted for 27.7% of the sequenced clones, which belonged primarily to Prymnesiophyceae. Unexpectedly, five operational taxonomic units of Cryptophyta were closely related to freshwater species. The Chlorophyta mostly fell within the Prasinophyceae, which was comprised of six clades, including Clade III, which is detected in the SCS for the first time in this study. Among the photosynthetic stramenopiles, Chrysophyceae was the most diverse taxon, which included seven clades. The majority of 18S rDNA sequences affiliated with the Dictyochophyceae, Eustigmatophyceae, and Pelagophyceae were closely related to those of pure cultures. The results of redundancy analysis and the permutation Mantel test based on unweighted UniFrac distances, conducted for spatial analyses of the Haptophyta subclades suggested that the Mekong River plume and cyclonic eddy play important roles in regulating microbial eukaryotic diversity and distribution in the southwestern SCS.

## Introduction

Microbial eukaryotes, pivotal components of marine ecosystems, are ubiquitous in the world ocean (de Vargas et al., 2015). Mesoscale features, such as river plumes and cyclonic eddies, are widespread in the global ocean and are critical components that enhance the efficiency of the biological pump (Oschlies and Garçon, 1998; Chelton et al., 2011; Kang et al., 2013). Cyclonic eddies in particular have been widely demonstrated to enhance nutrient inputs to the surface ocean and increase new production (Falkowski et al., 1991; Morán et al., 2001). However, although numerous studies have been conducted to determine particle flux in cyclonic eddies, and only a few focused on the phylogenetic diversity of planktonic organisms. Baltar et al. (2010) investigated prokaryotic assemblage structure and activity in mesoscale eddies in the Canary Current system, revealing that these eddies had distinct community compositions. In the Sargasso Sea, a distinguished phylogenetic difference in bacterioplankton has been clearly demonstrated by community profiling according to relative abundances of pyrosequenced amplicons (Nelson et al., 2014). Despite their important ecological roles (Massana, 2011; Caron et al., 2012), microbial eukaryotic diversity and distribution have been rarely reported in the cyclonic eddy-influenced ecosystem.

Among marine microbial eukaryotes, the pigmented taxa are extremely diverse and are recognized as important primary producers in open oceans (Li, 1994; Vaulot et al., 2008). However, the results of molecular surveys are commonly dominated by heterotrophs when polymerase chain reaction (PCR)-based approaches are used (Díez et al., 2001; Moon-van der Staay et al., 2001; Not et al., 2007; Stoeck et al., 2009). Methodological bias was even observed using novel flow cytometry sorting approaches (Marie et al., 2010), with the diversity of pigmented microbial eukaryotes typically being dominated by several taxa, including the Prymnesiophyceae, Mamiellophyceae, and Cryptophyceae, in sorted samples. The utility of molecular methods (e.g., 18S rDNA clone library construction and next-generation sequencing) is considered to be severely limited due to the use of universal primers to address photosynthetic microbial eukaryotic diversity.

From August to September 2007, a cyclonic eddy in the southwestern South China Sea (SCS) was well depicted (Hu et al., 2011), and its ecological characteristics were discussed (Chen et al., 2009; Zhang et al., 2011). Chen et al. (2010) reported the biogeochemical effects associated with the Mekong River plume in this area. Here, we further characterize the genetic diversity of microbial eukaryotes in these mesoscale process-influenced environments. We performed 18S rDNA cloning and sequencing associated with restriction fragment length polymorphisms (RFLPs), revealing an abundance of sequences affiliated with pigmented taxa in these mesoscale-influenced ecosystems for the first time. Furthermore, using redundancy analysis (RDA) and the permutation Mantel test, we analyzed the effects of mesoscale processes on the regulation of diversity and distribution of the major pigmented group Haptophyta. This study significantly expands upon the current knowledge of microbial eukaryotic diversity and distribution in these mesoscale process-influenced environments.

## Materials and Methods

### Sample collection

**Figure 1:**
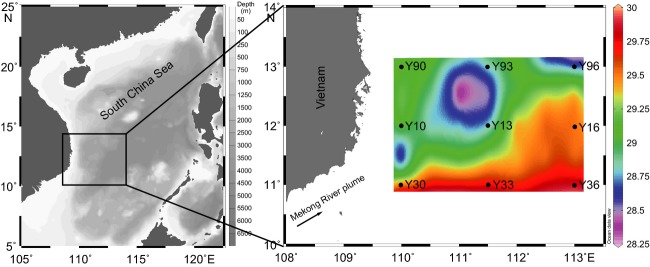
Sampling locations in the southwestern South China Sea (Schlitzer, 2014). Dots on the map correspond to the sampled stations for molecular analyses and pigment measurements. The colored background depicts the real-time surface water temperatures (°C) at all stations investigated during the cruise in 2007.

Sampling was conducted at nine stations in the southwestern SCS on board from September 2 through 8, 2007 (Fig.1). CTD casts equipped with an SBE-911 instrument (Sea-Bird) were used to obtain in situ vertical profiles of temperature, salinity and fluorescence. Irradiances were obtained from Moderate Resolution Imaging Spectroradiometer (MODIS) observation and an empirical formula (Morel, 1988). Mixed layer depth was defined as the first depth where the temperature was 0.2°C lower than that at surface (Steinhoff et al., 2010).

For molecular analyses, two samples were collected from surface water and the deep chlorophyll maximum (DCM) layer at each of the nine sampling stations. Briefly, after being filtered through a 10-*μ*m pore size mesh, 8–10-L seawater samples were prefiltered through 5-*μ*m pore size polycarbonate filters (Millipore, Ireland). Subsequently, the penetrated seawater samples were filtered through GF/F filters (Whatman, USA). Samples were immediately frozen in liquid nitrogen, brought back to the laboratory and stored at −80°C until analyses.

For pigment analyses, six samples were collected at various depths (surface, 25, 50, 75, 100, and 150 m). One of the six selected layers was replaced by the DCM at each station based on real-time hydrological and fluorescence profiles obtained from CTD sensors. Seawater samples of 4–16 L were directly filtered onto GF/F filters at a pressure of lower than 150 mm Hg. Samples were immediately frozen in liquid nitrogen on board and protected from light. Filters were then stored at −80°C in the laboratory until further processing.

Nutrient samples and pigment samples were collected together. Seawater samples were filtered through 0.45-*μ*m pore size acetate-fiber filters, and they were analyzed within 24 h on board.

### DNA extraction, cloning, and sequencing

Filters were thawed at room temperature, and DNA was extracted according to Countway et al. (2005) using the phenol:chloroform:isoamylalcohol (Sigma-Aldrich, USA) method. PCR and cloning were performed in two steps.

First, four universal eukaryotic primer sets (Table1 and Fig. S1) were selected to amplify the near-full-length 18S rDNA fragments of the DNA mixture containing the 18 samples. PCR amplification was carried out using a Go-*Taq* Flexi DNA Polymerase Kit (Promega, USA). Thermal cycling was performed with a thermocycler (Bio-Rad, Mexico) using the following protocol: one cycle at 95°C for 5 min, 30 cycles at 94°C for 40 sec, 55°C for 40 sec, 72°C for 1.5 min, and a final extension at 72°C for 10 min, followed by a hold at 4°C. For each of the four primer sets, three separate reactions were performed and the PCR products were pooled for construction of the following four clone libraries: WS071 (Euk328f+1498R), WS072 (EukA+1498R), WS073 (82F+1498R), and WS074 (Euk328f+Euk329r). Clone libraries were constructed using a TA cloning kit (TaKaRa, China), following the manufacturer's instructions. For each clone library, PCR amplification products of 400 positive clones were digested with the restriction enzymes *Hae*III and *Hha*I (TaKaRa) and run on a 3% agarose gel for RFLP analysis. For highly abundant RFLP patterns, 1–3 clones were sequenced with an ABI 3730xl system (Applied BioSystems, USA) to address the possibility that the patterns were present in different species (Lekunberri et al., 2014). Two sequencing reactions were performed for each selected clone to obtain an approximately 1800-bp fragment. For phylogenetic analyses, these sequences were re-grouped into operational taxonomic units (OTUs) based on 98% similarity.

**Table 1:** List of primers used in this study

Direction	Primer	Sequence (5′–3′)	Source
Forward	EukA	AACCTGGTTGATCCTGCCAGT	Medlin et al. (1988)
	Euk328f	ACCTGGTTGATCCTGCCAG	Moon-van der Staay et al. (2001)
	82F	GAAACTGCGAATGGCTC	López-García et al. (2003)
Reverse	EukB	TGATCCTTCTGCAGGTTCACCTAC	Medlin et al. (1988)
	Euk329r	TGATCCTTCYGCAGGTTCAC	Moon-van der Staay et al. (2001)
	1498R	CACCTACGGAAACCTTGTTA	López-García et al. (2003)

Second, PCR amplification was separately performed for each of the 18 samples using the primer set that showed the highest diversity among the four primer sets. Eighteen clone libraries were constructed following the above-mentioned procedures. For each clone library, 100 positive clones were randomly selected and partially sequenced in one sequencing reaction.

Nucleotide sequences identified in this study were deposited in GenBank database under the accession numbers KP404633–KP404914 (ca. 1800 bp) and KP404915–KP405223 (ca. 900 bp).

### Phylogenetic analyses

Sequences were analyzed with KeyDNAtools (www.keydnatools.com) for detecting chimeras. Partial fragments of putative chimera were then individually examined using BLAST (Zhang et al., 2000). Good-quality, near-full-length sequences identified with the four primer sets were used for the following phylogenetic analyses. Sequences aligned with MAFFT 7 (Katoh and Standley, 2013) were grouped into OTUs, as defined using Mothur v1.30.0 (Schloss et al., 2009), at 98% similarity. Venn diagrams were generated to compare the number of overlapping OTUs among the four clone libraries.

Subsets of sequences at different taxonomic levels were chosen based on assignment using PR^2^ database (Guillou et al., 2013). Sequences from each subset, together with their closest relatives, were realigned using MAFFT 7. Poorly aligned and divergent regions were eliminated using Gblocks v0.91b (Castresana, 2000), with a minimum block of five and allowed gap positions equal to half. Nest models of DNA substitution and associated parameters were estimated using jModelTest 2 (Darriba et al., 2012), and maximum likelihood (ML) phylogenetic trees were constructed using PhyML 3.1 (Guindon et al., 2010), with bootstrap analysis of 1000 replicates. Bayesian interferences were calculated with MrBayes v3.2.1 (Ronquist et al., 2012) using the GTR + G + I model and two runs of 1 million generations. The first 25% of the samples were discarded as ‘burn-ins’.

### Distribution patterns of Haptophyta subclades

Unique Haptophyta sequences (100% sequence similarity) obtained from the 18 separate clone libraries were used for spatial analyses. Relative contributions of different Haptophyta groups to the eukaryotic communities were calculated to identify distribution variations in the different environments. According to the relative abundances of Haptophyta phylotypes, the distribution patterns were illustrated by a heatmap coupled with an unweighted pair-group method with arithmetic mean (UPGMA) dendrogram of the 18 stations based on Bray-Curtis distances. RDA, combined with the ‘*envfit*’ function in vegan packages (Oksanen et al., 2013) was performed using R program (R Core Team 2014). The permutation Mantel test was used to estimate the correlations between the environmental distances and unweighted UniFrac distances (Lozupone and Knight, 2005) based on a neighbor-joining tree.

### Pigment and nutrient analyses

Pigment concentrations were determined using high-performance liquid chromatography (HPLC), according to previously decribed methods (Zapata et al., 2000; Mendes et al., 2007). Phytoplankton pigments were extracted using N,N-dimethylformamide (Furuya et al., 1998), and the extracts were then passed through GF/F filters of 13 mm in diameter to remove debris. For each sample, 600 *μ*L of extract was mixed with a 1 mol L^−1^ ammonium acetate solution of an equal volume. Pigment concentrations were determined with an Agilent series 1100 HPLC system and a 3.5-*μ*m Eclipse XDB C_8_ column (Agilent Technologies, USA). The analytical methods based on a gradient elution procedure were performed with a methanol:ammonium acetate (1 mol L^−1^) mixture (80:20) and a 100% methanol solution (solvents A and B, respectively). Standards supplied by DHI Water and Environment (Denmark) were used to identify and quantify of the following pigments: 19′-butanoyloxyfucoxanthin, 19′-hexanoyloxyfucoxanthin, alloxanthin, *α*-carotene, *β*-carotene, chlorophyll *a*, chlorophyllide *a*, chlorophyll *b*, chlorophyll *c*_1_ + chlorophyll *c*_2_, chlorophyll *c*_3_, diadinoxanthin, diatoxanthin, divinyl chlorophyll *a*, fucoxanthin, lutein, neoxanthin, peridinin, prasinoxanthin, violaxanthin, and zeaxanthin. The relative contributions of the different phytoplankton groups to the total chlorophyll *a* (Chl *a*) biomass were estimated using CHEMTAX matrix factorization (Mackey et al., 1996). Nine phytoplankton groups, including dinoflagellates, diatoms, haptophytes_8, haptophytes_6, chlorophytes, cryptophytes, prasinophytes, *Synechococcus*, and *Prochlorococcus*, were identified to be responsible for the total Chl *a* biomass (Goericke and Repeta, 1993; Latasa, 2007). The major differences between hyptophytes_8 and hyptophytes_6 were the distinct concentrations of 19′-butanoyloxyfucoxanthin (abundant in haptophytes_8) and monovinyl chlorophyll *c*_3_ (present in haptophytes_6; Zapata et al., 2004). Nutrient concentrations were analyzed using a Technicon AA3 Auto-Analyzer (Bran-Luebbe, Germany).

## Results and Discussion

### Hydrographic characteristics

**Figure 2:**
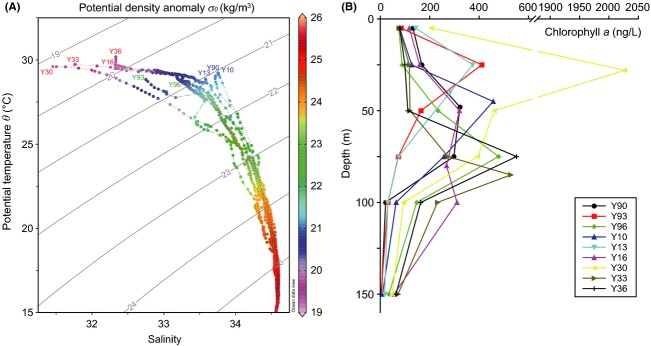
(A) T-S diagram of hydrographic stations and (B) vertical profiles of chlorophyll *a*.

A well-developed cyclonic eddy was identified in the southwestern SCS during the survey period (Fig.1), and its three-dimensional structure and physical properties were thoroughly characterized by Hu et al. (2011). Satellite altimeter data showed that the core of this eddy was stably located at 110.8°E, 12.2°N and that the horizontal measurements were approximately 60–90 km for 2 weeks, from August 26th to September 10th, 2007 (Hu et al., 2011). Accordingly, we found that all investigated stations in this study were influenced by the moving cyclonic eddy to some extent. The T-S diagram revealed vertical and horizontal variations in hydrography among the nine sampled stations (Fig.2A). Due to the strong influences of the plume, the highest Chl *a* concentration of 2026.9 ng L^−1^ was observed at a 28 m depth at station Y30 (Fig.2B). At the three stations (Y10, Y13, and Y90) characterized by lower surface temperatures, all of the DCM depths were shallower than 50 m, indicating stronger influences of the cyclonic eddy. However, Y33, Y36, and Y96 possessed deeper DCMs at 85 m (527.8 ng L^−1^) and 75 m (551.1 ng L^−1^, Y36; 478.6 ng L^−1^, Y96). The highest Chl *a* concentrations of Y93 and Y16 were 410.6 ng L^−1^ at 25 m and 267.9 ng L^−1^ at 50 m, respectively; however, we obtained samples at 50 m (Y93) and 80 m (Y16) with Chl *a* concentrations of 164.6 and 320.2 ng L^−1^.

### Community structures

In total, 282 near-full-length 18S rDNA sequences were obtained from the four clone libraries using mixed DNA templates and different primer sets. Remarkably, the majority of the sequences identified were associated with photosynthetic groups (Fig. S2). The Haptophyta was the most abundant taxon, accounting for 27.7% of the sequenced clones on average. Photosynthetic stramenopiles (including the Bacillariophyceae, Pelagophyceae, Chrysophyceae, and Dictyochophyceae) contributed an average of 23.4% to the total recovered sequences. Sequences belonging to heterotrophs accounted for only 17.4% of the total sequenced clones. Notably, only 6 OTUs were shared by the four clone libraries (Fig. S3). Primer bias is widely known as one of the main shortcomings of PCR-based diversity surveys (Potvin and Lovejoy, 2009). The use of multiple primer sets has been suggested as an approach for improving the recovery of protistan sequences (Stoeck et al., 2006). This method also appears to be essential for photosynthetic microbial eukaryotes, according to the results of this study. For example, the primer set 82F and 1498R amplified all recovered Cryptophyta sequences, but Dictyochophyceae sequences were only detected in the clone library using Euk328f and Euk329r. To elucidate how mesoscale processes regulate the major microbial eukaryotic groups, particular focus was placed on the Haptophyta. The primer set 82F+1498R, which detected the greatest number of OTUs (97 OTUs), was selected for the following PCR amplifications of the 18 separate samples.

### Photosynthetic microbial eukaryotic diversity

**Figure 3:**
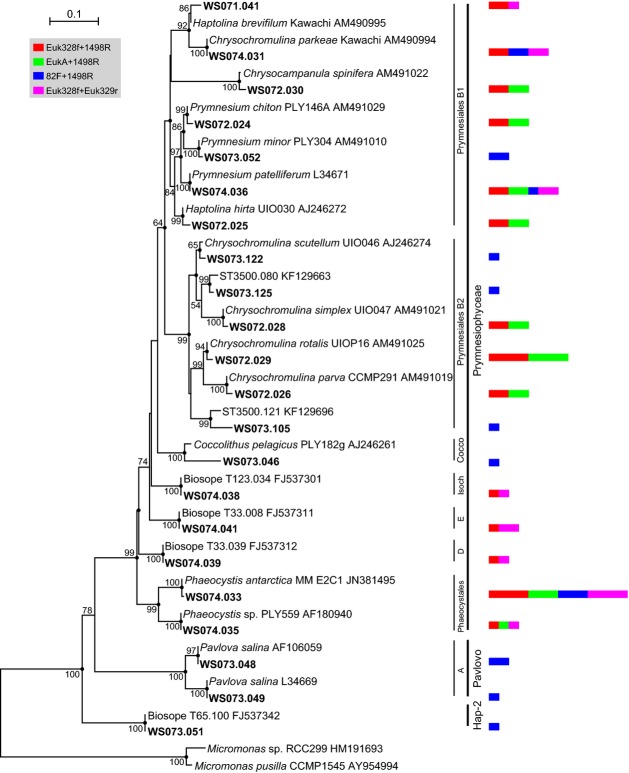
Phylogenetic tree for Haptophyta based on maximum likelihood analysis of 18S rDNA sequences (1696 positions) retrieved from the southwestern South China Sea (bold). The tree is based on a TrNef + G + I model of DNA substitution with a gamma distribution shape parameter of 0.495 (I = 0.477), and R(b)[A–G] = 1.9121, R(e)[C–T] = 4.0299 and 1.0 for the other substitution rates. Bootstrap values of >50% (1000 replicates) are shown, and the filled circles at the nodes indicate Bayesian posterior probabilities of >0.9. The tree is rooted by two *Micromonas* sequences. The multi-value bar charts correspond to the number of clones represented by each operational taxonomic unit. The scale bars indicate 0.1 substitutions per base (black line) and one clone (color bar chart). Cocco, Coccolithales; Isoch, Isochrysidales; Pavlovo, Pavlovophyceae.

Sequences affiliated with the Haptophyta belonged primarily to Prymnesiophyceae (Fig.3). Of the seven detected Prymnesiophyceae clades, Prymnsiales B1 and B2 were characterized by higher diversity. Previously, we conducted a relatively complete survey of the SCS, ranging from the southernmost Nansha Islands to the northernmost coast (Wu et al., 2014a), and the Haptophyta was found to be the most diverse group of photosynthetic microbial eukaryotes. Surprisingly, sequences affiliated with the order Prymnesiales B1 that were frequently detected in this area have been rarely reported in previous surveys of the SCS (Yuan et al., 2004; Cheung et al., 2008). Coccolithales and Clade E, represented by the clones WS073.046 and WS074.041, respectively, were detected for the first time in the SCS in this study. Notably, clones of Clade E, Clade D (WS074.039) and Isochrysidales (WS074.038) were closely related to environmental sequences detected from the oligotrophic southeastern Pacific Ocean, with sequence similarities of 100%. One OTU affiliated with the Phaeocystales possessed the greatest number of clones, totaling 14, and was represented by WS074.033. Clone WS073.051 was affiliated with the recently established Hap-2, sharing 100% sequence similarity with Biosope T65.100, supporting the existence of deep-branching novel lineages and high diversity of the Haptophyta in the subtropical–tropical environment (Egge et al., 2015a; Egge et al., 2015b).

**Figure 4:**
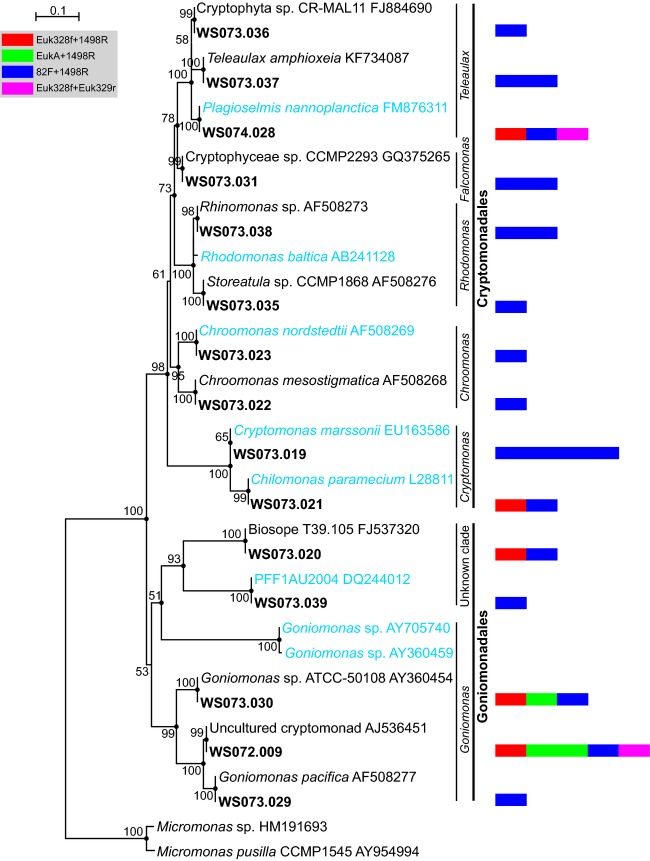
Phylogenetic tree for Cryptophyta based on maximum likelihood analysis of 18S rDNA sequences (1615 positions) retrieved from the southwestern South China Sea (bold). The tree is based on a TrN + G + I model of DNA substitution with a gamma distribution shape parameter of 0.67 (I = 0.49), and R(b)[A–G] = 2.2363, R(e)[C–T] = 4.557 and 1.0 for the other substitution rates. Bootstrap values of >50% (1000 replicates) are shown, and the filled circles at the nodes indicate Bayesian posterior probabilities of >0.9. The tree is rooted by two *Micromonas* sequences, and sequences that originated from freshwater are marked in cyan. The multi-value bar charts correspond to the number of clones represented by each operational taxonomic unit. The scale bars indicate 0.1 substitutions per base (black line) and one clone (color bar chart).

Thirty-one Cryptophyta sequences were identified and were grouped into 15 OTUs. Surprisingly, 5 OTUs were closely related to freshwater species (Fig.4). Specifically, three of the five detected Cryptomonadales genera (*Teleaulax*, *Chroomonas* and *Cryptomonas*) contained sequences that shared 100% similarity with 18S rDNA sequences from freshwater organisms. Such a large number of 18S rDNA sequences related to freshwater species has not been previously reported in marine environments. Cryptophyta is well known to be extremely diverse in freshwater environments, and some taxa, such as *Cryptomonas* are widespread in lakes (Eiler et al., 2013). Goes et al. (2014) have reported the sporadic presence of Cryptophyta in the Amazon River estuary and in a plume in the western tropical North Atlantic. High abundances and biomass have been frequently reported in the Amazon River estuary; however, strikingly small biomass contributions of the Cryptophyta have been observed in the river plume far from the Amazon River estuary. Similarly, higher biomass contributions were observed in the DCMs of the stations with lower salinity levels in this study (Fig. S4), and the contributions of the Cryptophyta were decreased accordingly with increasing distances from the Mekong River estuary (e.g., 3.31, 3.29 and 1.87% in the DCMs of Y30, Y33 and Y36, respectively). It is reasonable to hypothesize that the Cryptophyta sequences that we detected originated from species transported by the Mekong River plume. Furthermore, it could also be inferred from our results that these Cryptophyta species survive not only in freshwater systems but also in marine environments. Among the order Goniomonadales, nine sequences (3 OTUs) affiliated with the genus *Goniomonas* were closely related to seawater species, with sequence similarities of above 99%. Additionally, two OTUs represented by WS073.020 and WS073.039 were unknown clades that shared 100% similarity with their references, Biosope T39.105 and PFF1AU2004, respectively.

**Figure 5:**
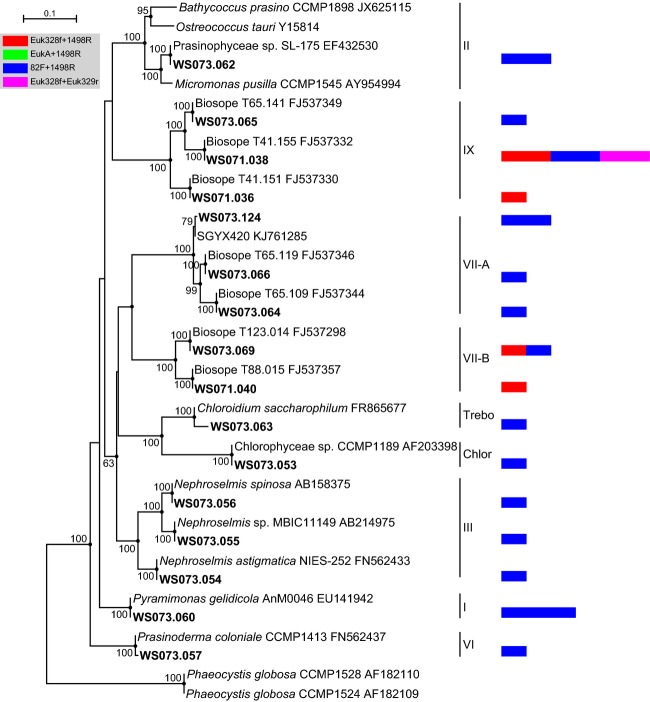
Phylogenetic tree for Chlorophyta based on maximum likelihood analysis of 18S rDNA sequences (1680 positions) retrieved from the southwestern South China Sea (bold). The tree is based on a TrNef + G + I model of DNA substitution with a gamma distribution shape parameter of 0.534 (I = 0.313), and R(b)[A–G] = 2.5138, R(e)[C–T] = 4.4253 and 1.0 for the other substitution rates. Bootstrap values of >50% (1000 replicates) are shown, and the filled circles at the nodes indicate Bayesian posterior probabilities of >0.9. The tree is rooted by two *Phaeocystis* sequences. The multivalue bar charts correspond to the number of clones represented by each operational taxonomic unit. The scale bars indicate 0.1 substitutions per base (black line) and one clone (color bar chart). Trebo, Trebouxiophyceae; Chlor, Chlorophyceae.

The Chlorophyta fell primarily within the Prasinophyceae, which was comprised of six clades, Clades I, III, VI, VII, and IX and Mamiellophyceae (Fig.5). Clade III identified in this study is a novel clade that has not been previously reported in the SCS (Yuan et al., 2004; Cheung et al., 2008; Wu et al., 2014a). Among the Chlorophyta, sequences affiliated with *Micromonas*, *Ostreococcus*, and *Bathycoccus* are ubiquitous and are commonly detected in various habitats. Surprisingly, no sequences of these three genera of Mamiellophyceae were recovered here. In previous studies of other seas, Clade IX has been identified only in flow cytometry-sorted samples or using a phylum-biased approach (Viprey et al., 2008; Shi et al., 2009). Our frequent identification of Clade IX sequences in filtered samples from the SCS using universal primers was unexpected (Wu et al., 2014a; Wu et al., 2014b). The limited knowledge of Clade IX highlights the need for increased culture isolation efforts to characterize common species in molecular studies (Pedrós-Alió, 2006).

**Figure 6:**
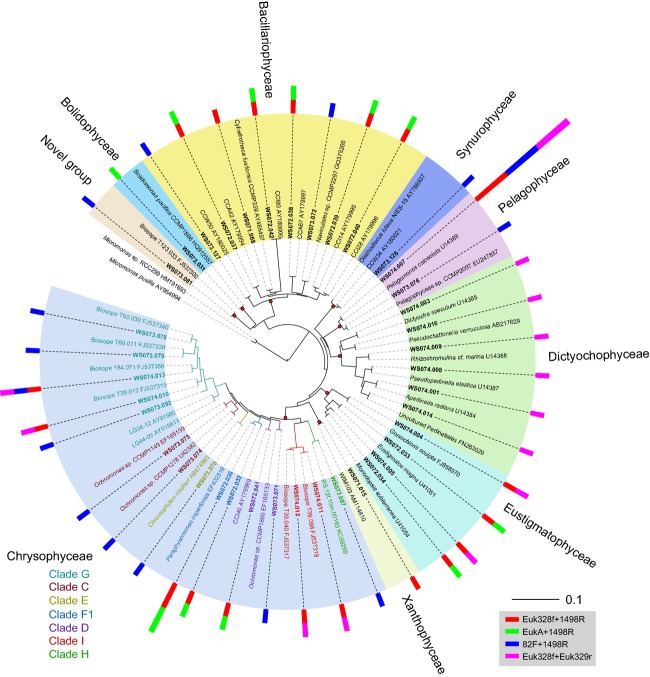
Phylogenetic tree for photosynthetic stramenopiles based on maximum likelihood analysis of 18S rDNA sequences (1657 positions) retrieved from the southwestern South China Sea (bold). The tree is based on a TrN + G + I model of DNA substitution with a gamma distribution shape parameter of 0.502 (I = 0.359), and R(b)[A–G] = 2.3457, R(e)[C–T] = 4.3974 and 1.0 for the other substitution rates. The red dots at the nodes indicate a bootstrap value of 100% (1000 replicates) and a Bayesian posterior probability of 1.0. The tree is rooted by two *Micromonas* sequences. The multivalue bar charts correspond to the number of clones represented by each operational taxonomic unit. The scale bars indicate 0.1 substitutions per base (black line) and one clone (color bar chart).

The photosynthetic stramenopiles were the most diverse groups, containing 66 sequences grouped into 39 OTUs (Fig.6). Among the Chrysophyceae, sequences of Clades G and I shared high similarities of above 99% with environmental references detected in the southeastern Pacific Ocean, except for WS073.092. A number of Bacillariophyceae sequences were detected in these size-fractioned samples. The clone WS073.081 clustered with Biosope T123.033 as a novel group sharing 100% similarity. Notably, the similarity of WS073.081 to the second most closely related sequence in GenBank database dropped sharply to 91%. The majority of sequences affiliated with the Dictyochophyceae, Eustigmatophyceae, and Pelagophyceae were the most closely matched to sequences from cultures. One clone was recovered for each of Xanthophyceae, Synurophyceae, and Bolidophyceae. Generally, Dictyochophyceae, Eustigmatophyceae Xanthophyceae, Synurophyceae, and Bolidophyceae are rarely identified in 18S rDNA clone libraries and seem to be ‘rare species’ in molecular surveys. In fact, numerous 18S rDNA sequences from pure cultures have proven difficult to detect in molecular surveys, and the reason for this difficulty has been primarily attributed to culture bias (del Campo et al., 2014). Our results demonstrate the recovery of numerous environmental sequences from these taxa in these mesoscale process-influenced systems, providing new insights into the diversity and distribution of these organisms.

### Haptophyta communities in relation to environmental variables

**Figure 7:**
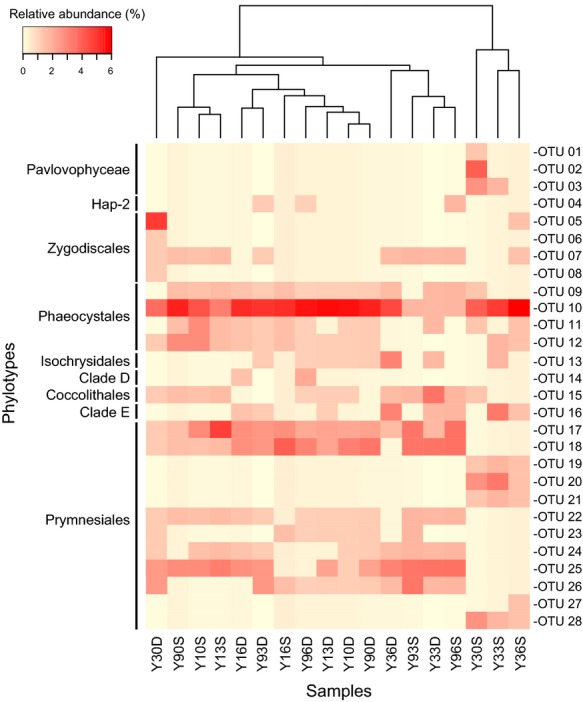
Hierarchical heatmap showing distribution patterns based on the relative abundances of unique Haptophyta phylotypes (100% sequence similarity), coupled with a unweighted pair-group method with arithmetic mean dendrogram of 18 samples constructed based on Bray-Curtis distances.

The separate clone libraries using the primer set 82F+1498R were found to contain abundant Haptophyta lineages and they were extremely similar to the taxa detected using the four different primer sets (Fig.7). A total of 309 clones affiliated with the Haptophyta were detected and included 28 unique phylotypes (100% sequence similarity). One OTU (OTU 10) affiliated with the Phaeocystales (*Phaeocystis antarctica*) was detected in each clone library with a high relative contribution of a maximum of 6%. Based on Bray-Curtis distances, the samples collected from locations significantly influenced by the Mekong River plume (Y30S, Y33S and Y36S) were separated from the others. Surface (Y10S, Y13S, and Y90S) and DCM samples (Y10D, Y13D, and Y90D) collected from the cyclonic eddy-influenced station were also clustered together, suggesting the important role of mesoscale processes in regulating Haptophyta population structures.

**Figure 8:**
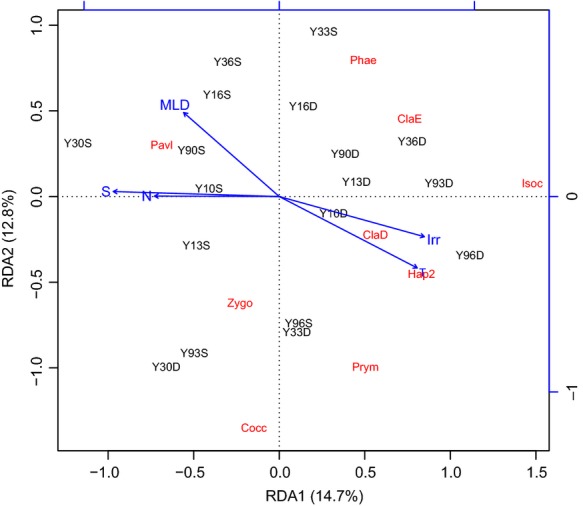
Ordination diagram of the first two axes of redundancy analysis, showing the relationships between the relative abundances of Haptophyta subclades and environmental variables. T, temperature; S, salinity; Irr, irradiance; MLD, mixed layer depth; N, NO_2_ + NO_3_; Cocc, Coccolithales; Isoc, Isochrysidales; ClaD, Clade D; ClaE, Clade E; Pavl, Pavlovophyceae; Phae, Phaeocystales; Prym, Prymnesiales; Hap2, Hap-2; Zygo, Zygodiscales.

An RDA plot was generated based on the environmental parameters and relative abundances of Haptophyta phylotypes, showing that 27.5% of the variability could be explained by the environmental parameters (Fig.8). In addition, the Mantel test based on unweighted UniFrac distances suggested that environmental variables played important roles in shaping the communities of Haptophyta subclades (*P *<* *0.05, permutations = 999). RDA further suggested that changes in community composition were positively correlated with salinity, nutrients and Chl *a* concentrations in surface samples, in contrast with the DCMs, in which community shifts were positively correlated with temperature and irradiance. Negative clustering by temperature indicated that Y10S, Y13S, and Y90S were more strongly influenced by the cyclonic eddy in the shaping of the Haptophyta community. The distribution of Pavlovophyceae showed strong correlations with the nutrient concentrations and the sample Y30S, which was affected by the Mekong River plume. As revealed in Fig.3, the Pavlovophyceae sequences were affiliated with *Pavlova salina* (L34669), which has undergone adaptation to brackish environments (Bendif et al., 2011). Clade D, Clade E, Hap-2, and the Isochrysidales appeared to be predominant in the DCM samples. The Coccolithales and Zygodiscales were located in the corner and center of the RDA plot, indicating that there were no significantly close relationships between their distributions and the environmental variables. No single environmental variable was found to be responsible for the distribution of the Prymnesiales due to their widespread dispersal. Special traits of the Prymnesiales, such as mixotrophy might contribute to their adaptability to diverse environments (Unrein et al., 2014). Pigment analyses showed that haptophytes_8 were the most important eukaryotic group, contributing an average of 9.3% to the Chl *a* biomass (Fig. S4). Particularly in the DCMs, haptophytes_8 was the dominant group, with Chl *a* biomass contributions ranging from 9% (Y30) to 67.1% (Y10). Haptophytes_8 primarily included species affiliated with the order Phaeocystales (e.g., *Phaeocystis antarctica*, *Phaeocystis pouchetii* and *Phaeocystis globosa*) and was characterized by an abundance of 19′-butanoyloxyfucoxanthin (Zapata et al., 2004). This finding was confirmed by molecular analysis, which revealed an abundance of Phaeocystales sequences (Figs.3 and 7). Due to the frequent recovery of Phaeocystales in distinct environments, no parameter was determined to be responsible for their distribution patterns in the RDA plot.

## Conclusions

In summary, our study expands upon previous similar studies, reporting microbial eukaryotic communities characterized by abundant pigmented taxa in a river plume and cold eddy-influenced ecosystem. Undoubtedly, phylogenetic analyses based on numerous sequences provide important background information on these vital primary producers that have been missed in previous environmental surveys. Our study also has revealed that mesoscale processes have substantial effects on the diversity and distribution of microbial eukaryotes. To identify how mesoscale processes shape microbial eukaryotic communities in detail and how microbial eukaryotic diversity responds to mesoscale processes, more systematical studies are needed in the future.

## Supporting Information

**Figure S1.** Localization of the primers used in this study. The environmental sequence OLI11115 (GenBank accession number AJ402326) is used as a reference. **Figure S2.** Relative abundances of microbial eukaryotic groups in the four clone libraries using mixed DNA and different primer sets (Euk328f+1498R, EukA+1498R, 82F+1498R, and Euk328f+Euk329r). **Figure S3.** Venn diagram illustrating the operational taxonomic units for the clone libraries determined using four primer sets. **Figure S4.** The phytoplankton communities at each station with the percent contributions of the nine main groups to the total chlorophyll *a* biomass at the surface and the deep chlorophyll maximum (DCM).
