# Supplementary material for: Microbial eukaryotic diversity and distribution in a river plume and cyclonic eddy-influenced ecosystem in the South China Sea
Source: Microbiologyopen. 2015 Aug 12;4(5):826–40. doi: 10.1002/mbo3.282 (PMC4618614; doi:10.1002/mbo3.282)

**Supporting information**

**Figure S1.** Localization of the primers used in this study. The environmental sequence OLI11115 (GenBank accession number AJ402326) is used as a reference.


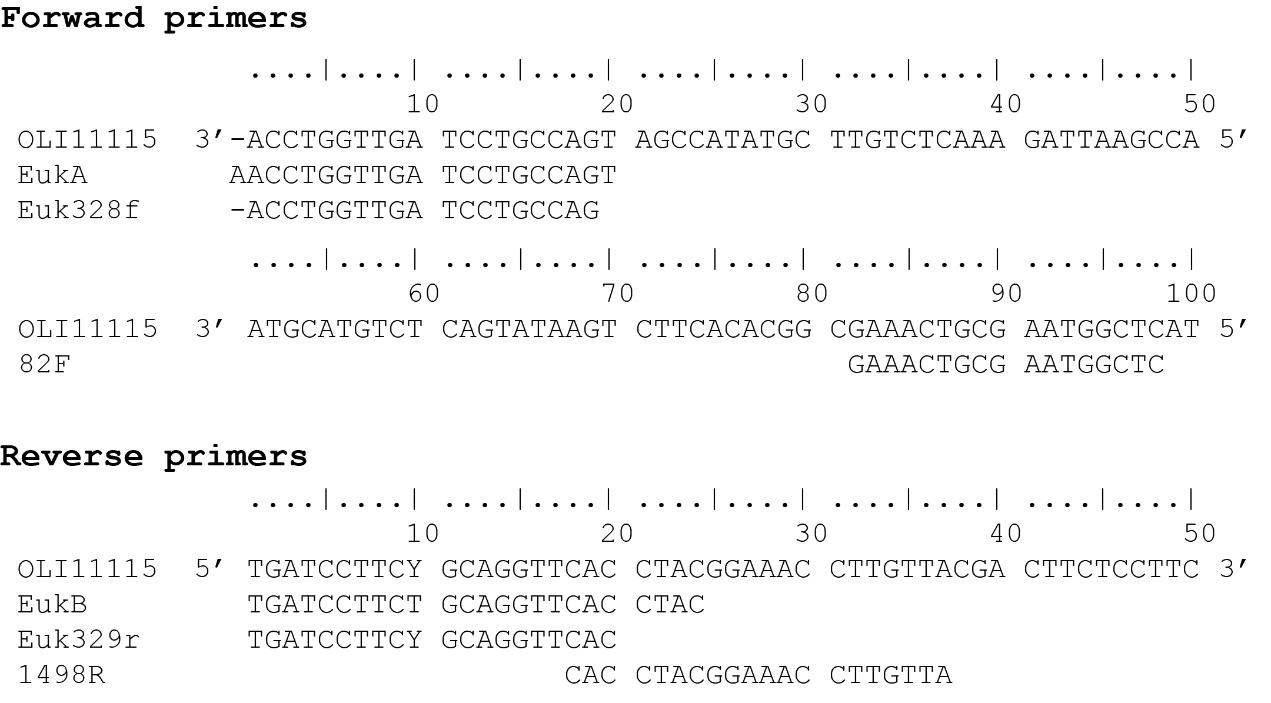


**Figure S2.** Relative abundances of microbial eukaryotic groups in the four clone libraries using mixed DNA and different primer sets (Euk328f+1498R, EukA+1498R, 82F+1498R and Euk328f+Euk329r). The numbers in the brackets are the numbers of sequences, and the pattern resulting from inclusion of 282 pooled sequences is also provided. PhotoStramenopiles = photosynthetic stramenopiles.


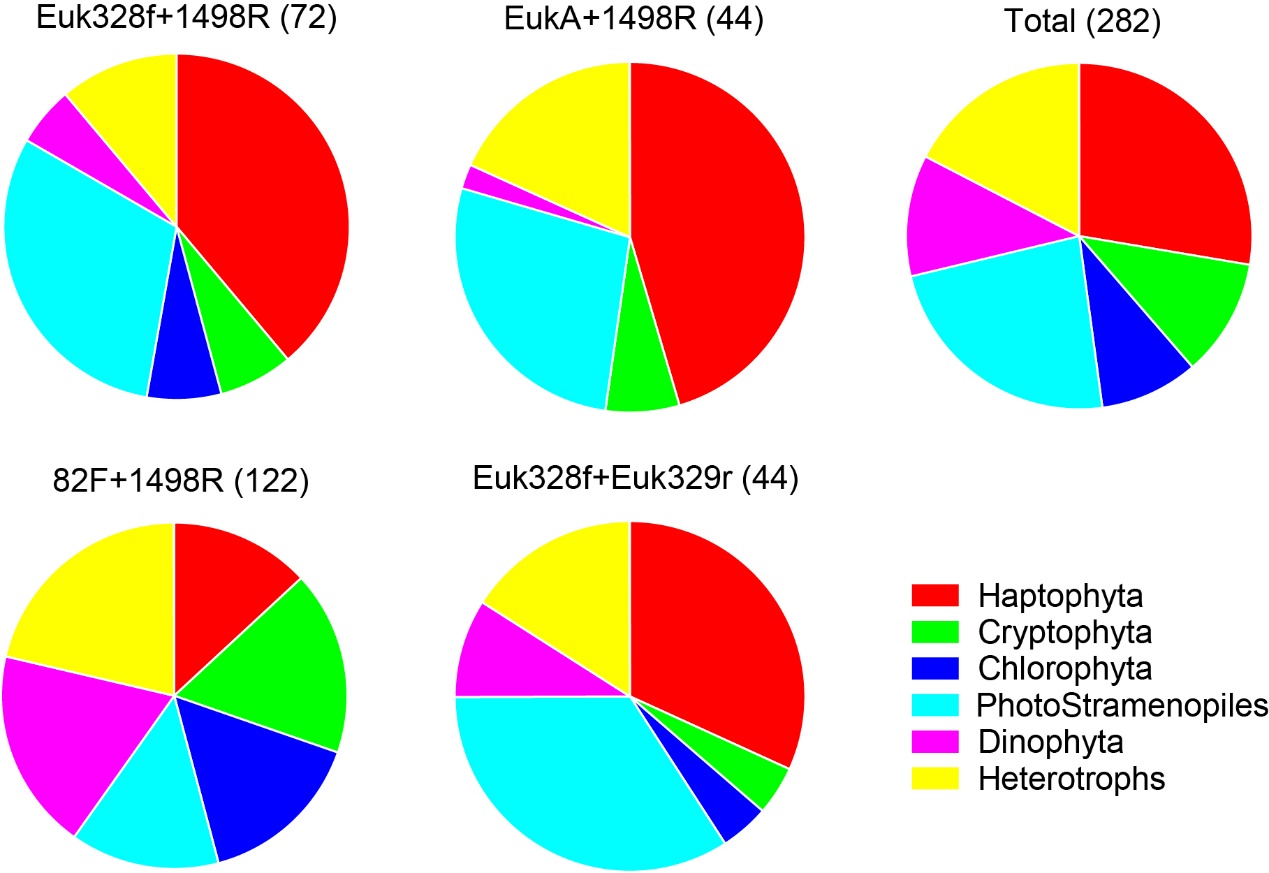


**Figure S3.** Venn diagram illustrating the operational taxonomic units for the clone libraries determined using four primer sets.

**
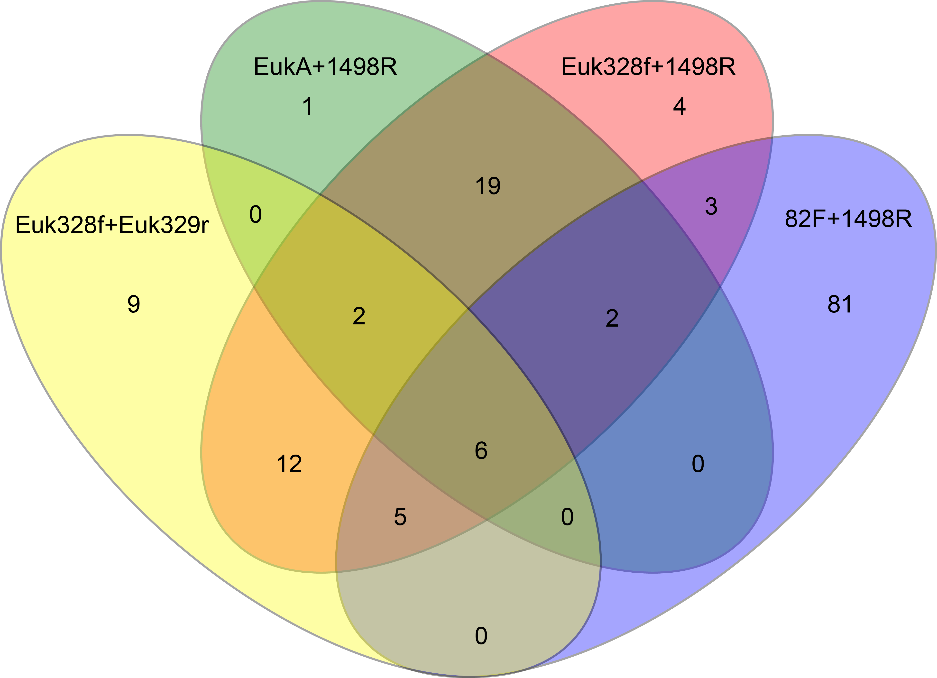
**

**Figure S4.** The phytoplankton communities at each station with the percent contributions of the 9 main groups to the total chlorophyll *a* biomass at the surface and the deep chlorophyll maximum (DCM).


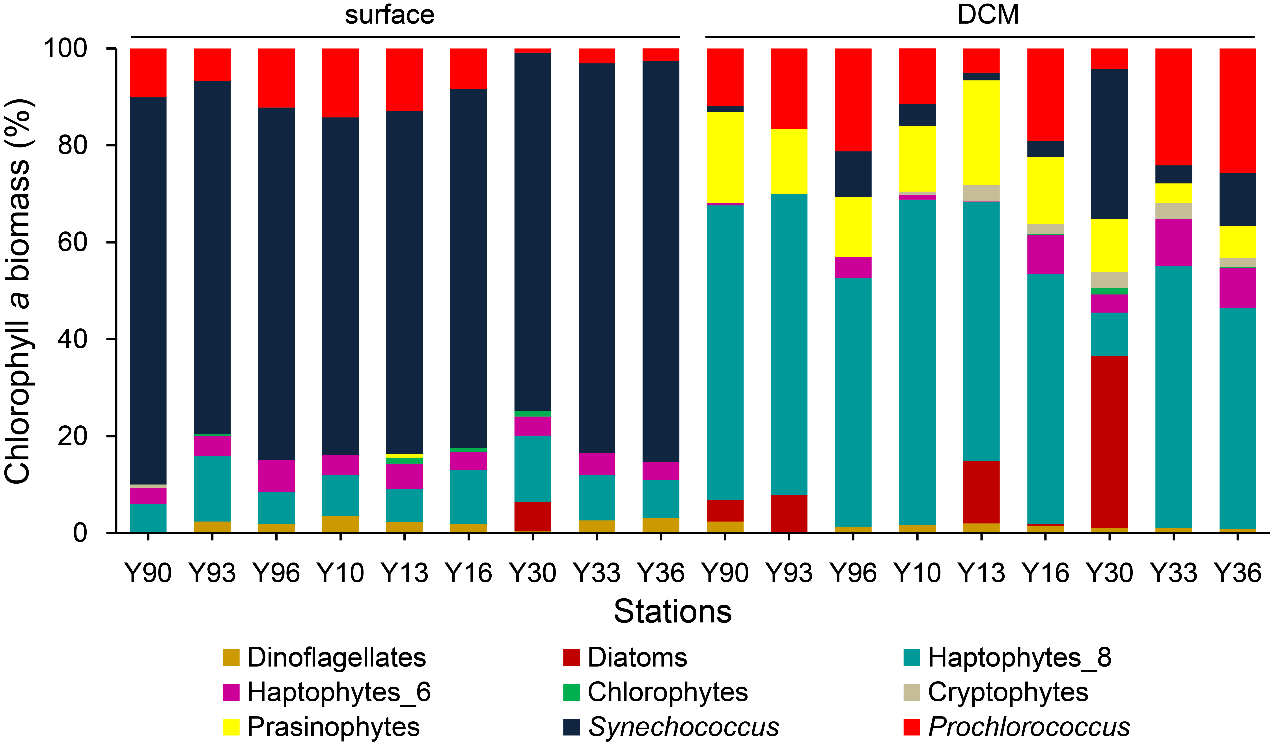

Supplement: Supplementary file 1 — Figure S1. Localization of the primers used in this study. The environmental sequence OLI11115 (GenBank accession number AJ402326) is used as a reference. Figure S2. Relative abundances of microbial eukaryotic groups in the four clone libraries using mixed DNA and different primer sets (Euk328f+1498R, EukA+1498R, 82F+1498R, and Euk328f+Euk329r). Figure S3. Venn diagram illustrating the operational taxonomic units for the clone libraries determined using four primer sets. Figure S4. The phytoplankton communities at each station with the percent contributions of the nine main groups to the total chlorophyll a biomass at the surface and the deep chlorophyll maximum (DCM). [file mbo30004-0826-sd1.docx]
